# Diagnostic Value of Aortic Dissection Risk Score, Coagulation Function, and Laboratory Indexes in Acute Aortic Dissection

**DOI:** 10.1155/2022/7447230

**Published:** 2022-04-19

**Authors:** Renjie Song, Nana Xu, Lan Luo, Tianxi Zhang, Haizhen Duan

**Affiliations:** Department of Emergency Medicine Affiliated Hospital of Zunyi Medical University, China

## Abstract

**Objective:**

This study was aimed at studying the diagnostic value of aortic dissection (AD) risk score, coagulation function, and laboratory indicators in acute aortic dissection (AAD).

**Methods:**

In this retrospective study, 57 patients with AAD and 57 with an acute coronary syndrome (ACS). During the same period, 50 healthy subjects were selected as the control group admitted to our institution which were assessed for eligibility and recruited. They were assigned to an AD group (AAD patients) and an ACS group (ACS patients). The AD risk scores, coagulation function indexes, and laboratory indexes of the two groups were compared. With digital subtraction angiography- (DSA-) based diagnosis result as the gold standard, the receiver operating characteristic (ROC) curve was used to analyze the diagnostic value of various indexes for AD, and the sensitivity, specificity, and optimal diagnostic value (Youden index) of the diagnostic indexes were calculated. Additionally, the overall blood clot formation strength (MA), clotting factor function (R), platelet function (MAp), and functional fibrinogen (MAf) levels were evaluated.

**Results:**

AAD risk, AD screening, early diagnosis of AAD, fibrinogen degradation products (FDP), fibrinogen (Fib), prothrombin time (PT), activated partial thromboplastin time (APTT), tenascin C (TN-C), D-dimer (D-D), and N-terminal B-type natriuretic peptide precursor (NT-proBNP) in the three groups were statistically different (*P* < 0.05). Further pairwise comparisons showed that the AD patients got higher scores of AAD risk, AD screening, and early diagnosis of AAD versus ACS patients (*P* < 0.05). AD was associated with lower levels of fibrinogen degradation products (FDP) and fibrinogen (Fib), shorter prothrombin time (PT), and activated partial thromboplastin time (APTT) versus ACS (*P* < 0.05). AD also resulted in higher levels of tenascin C (TN-C), D-dimer (D-D), and N-terminal B-type natriuretic peptide precursor (NT-proBNP) versus ACS (*P* < 0.05). The three risk scores, various laboratory indicators, and various coagulation function indicators were of high diagnostic values for the diagnosis of AAD (*AUC* > 0.9, *P* < 0.05). The sensitivity of the AD screening scale and TN-C expression level to the diagnosis of AAD was up to 100%, and the specificity of TN-C expression level was up to 98.25%. The influencing factors of AAD included Fib, FDP, PT, APTT, D-D, TN-C, and NT-proBNP. MA, MAf, and MAp displayed the same trend and reached the lowest point at T2. R was the opposite and reached the highest point at T2. At T4, a higher Map and a lower MAf were found than before surgery, and R and MA returned to preoperative levels. The positive detection rate of ACS by CT scan was positively correlated with the degree of stenosis (*r* = 0.814, *P* < 0.05).

**Conclusion:**

AD screening scale, TN-C, and FDP are of the highest diagnostic value in the risk score of AD, laboratory indicators, and coagulation function. It has implications for the diagnosis of ADD.

## 1. Introduction

Acute aortic dissection (AAD) is a critical illness of the cardiovascular system and is characterized by rapid progression and lethal symptoms [1, 2]. Patients' health and quality of life are severely compromised by its high mortality and poor prognosis, which consequently entails early diagnosis and timely treatment [3–5]. Currently, the digital subtraction angiography (DSA) results are the gold standard in the diagnosis of AAD and are used in clinical stent positioning and evaluation of endovascular treatment [6–8]. AD risk score is a commonly used early screening method in clinical practice. In recent years, laboratory indicators including tenascin C (TN-C), D-dimer (D-D), N-terminal B-type natriuretic peptide precursor (NT-proBNP), and coagulation function used in the auxiliary diagnosis of AAD have demonstrated a tremendous fascination on the academia. Therefore, this study was aimed at assessing the diagnostic value of the aforementioned indicators for AAD through a retrospective analysis.

## 2. Materials and Methods

### 2.1. General Data

Patients with acute coronary syndrome (ACS) and AAD were recruited according to diagnostic criteria: acute AD- and ACS-related criteria in *Internal Medicine* [9]. The inclusion criteria were as follows: (1) patients who met the acute AD- and ACS-related criteria in *Internal Medicine*; (2) patients with back pain, chest pain, or aortic regurgitation and without peripheral pulse or pulse unequal, shock, or hypertension; (3) with widened mediastinum or aorta and tear-induced intimal valve in intima of the aorta according to chest imaging; (4) with at least one coronary artery obstructed by ≥75% according to medical imaging for the chest, abnormal ST segment according to ECG, and abnormal myocardial enzyme examination results; (5) with confirmed diagnosis by DSA; and (6)with subsequent treatment in this hospital. The exclusion criteria were as follows: (1) AD or ACS patients with incomplete examination and treatment data; (2) with hospital referral; (3) with other types of acute cardiovascular disease; (4) with a history of cerebrovascular adverse events or acute pulmonary embolism within a month prior to this treatment; (5) with acute infectious disease, major surgery, or trauma within recent 14 days; and (6) with malignant tumors. In this retrospective study, 57 patients with AAD and 57 with an acute coronary syndrome (ACS) admitted to our institution were finally assessed for eligibility and recruited. They were assigned to an AD group (AAD patients) and an ACS group (ACS patients). During the same period, 50 healthy patients were selected as the control group.

### 2.2. Ethical Statement

The study was approved by the Hospital Ethics Committee, and the patients and their families were informed of the purpose and process of the study and signed the informed consent.

### 2.3. Methods

#### 2.3.1. AD Risk Score

The AD risk score of all patients was graded using the AAD risk score scale developed by the *American Heart Association*, AD screening sheet developed in Chinese expert consensus from the standardized assessment and diagnosis of chest pain (2014 version), and AAD early diagnosis score scale [10]. The AAD risk score scale included 6 items, with a total score of 13 points, a score of 1 to 4 points for a low risk of AAD, a score of 5 to 8 points for a moderate risk of AAD, and a score of >8 points for a high risk of AAD. The AD screening scale included 3 items, with a full score of 3 points, a score of 0 points for a low risk, a score of 1 point for a moderate risk, and a score of 2 to 3 points for a high risk. The AAD early diagnosis score scale included 5 items, with a total score of 13 points, a score >4 points for a high risk, and a score <4 points for a low risk.

#### 2.3.2. Laboratory Indicators and Coagulation Function Indicators

Laboratory indicators included TN-C, D-D, and NT-proBNP, and coagulation indicators included fibrinogen degradation products (FDP), fibrinogen (Fib), prothrombin time (PT), and activated partial thromboplastin time (APTT).

During assay, a total of 6 ml peripheral cubital venous blood were collected from all eligible patients and stored in 2 blood collection tubes, 3 ml in each. 3 ml blood was added with sodium citrate for anticoagulation, centrifuged at 4000 r/min for 8 min to obtain the plasma. The latex enhanced immune turbidimetric method was used to determine the DD level, the immunoenzyme-linked adsorption method was used to determine the TN-C level, and the automatic blood coagulometer and its supporting kit were used to determine the coagulation function indicators. The other 3 ml blood sample was centrifuged at 3000 r/min for 10 min to obtain the serum, and the level of NT-proBNP was determined by the electrochemiluminescence method.

### 2.4. Outcome Measures

The three AD risk scores, coagulation function indicators, and laboratory indicators between the two groups of patients were compared, and the sensitivity, specificity, and optimal cut-off value (Youden index) of diagnostic value were calculated using the DSA-based diagnosis result as the gold standard. Additionally, clinical data was used to analyze independent risk factors for coagulation dysfunction in patients after surgery.

According to the medical records, patients' blood samples (HEMO) at four time points (T1: after anesthesia; T2: during operation; T3: after drug treatment; and T4: at 4 h after medical treatment) were used to evaluate their overall blood clot strength (MA), coagulation factor function (R), platelet function (MAp), and functional fibrinogen level (MAf).

### 2.5. Statistical Analysis

All data analyses were performed using SPSS 23.0 software. The measurement data were expressed as $\bar{x}\pm s$ and analyzed by the *t* test; one-way ANOVA was used for comparison among multiple groups, and LSD *t* test was used for further pairwise comparisons. The Pearson analysis was used for correlation analysis. Receiver operating characteristic (ROC) curves were plotted to analyze the diagnostic value of various indicators for AD by calculating the area under the curve (AUC): AUC > 0.9 suggested a high diagnostic value, 0.7 < AUC ≤ 0.9 suggested a medium diagnostic value, 0.5 ≤ AUC ≤ 0.7 suggested a low diagnostic value, and AUC < 0.5 suggested no diagnostic value basically. Statistical significance was set at a *P* value of 0.05 or lower.

## 3. Results

### 3.1. Baseline Data

The baseline features of the ACS group (manifesting as unstable angina pectoris, non-ST-segment elevation acute myocardial infarction, NSTEM, and ST-segment elevation acute myocardial infarction) (41 males, 16 females, aged 48 to 76 years, mean age 63.07 ± 7.62 years, 46 cases of hypertension, 11 cases of diabetes, 29 cases of hyperlipidemia, 27 cases of smoking history, and 33 cases of drinking history) were comparable with those of the AD group (manifesting as severe pain, shock, and compression symptoms and cardiac tamponade, massive bleeding, malignant hypertension in a small number of patients) (45 males, 12 females, aged 46 to 75 years, mean age 63.12 ± 7.81 years, 48 cases of hypertension, 10 cases of diabetes, and 27 cases of hyperlipidemia and 29 cases of smoking history, and 31 cases of drinking history) and the control group (30 males, 20 females, aged 46 to 76 years, mean age 63.44 ± 6.91 years) (*P* > 0.05).

### 3.2. Risk Scores

AAD risk, AD screening, and early diagnosis of AAD in the three groups were statistically different (*P* < 0.05). Further pairwise comparisons showed that remarkably higher scores of AAD risk, AD screening, and early diagnosis of AAD were witnessed in the AD patients versus ACS patients (*P* < 0.05). (Table 1).

### 3.3. Coagulation Function Indexes

There were statistically significant differences in Fib, FDP, PT, and APTT among the three groups of patients (*P* < 0.05). Further pairwise comparisons revealed that the AD patients showed markedly higher levels of Fib, FDP, PT, and APTT versus ACS patients (*P* < 0.05). (Table 2).

### 3.4. Laboratory Indicators

There were statistically significant differences in D-D, TN-C, and NT-proBNP among the three groups (*P* < 0.05); further pairwise comparison revealed that the AD patients showed higher levels of D-D, TN-C, and NT-proBNP than the ACS patients (Table 3).

### 3.5. Diagnostic Value

**Figure 1:**
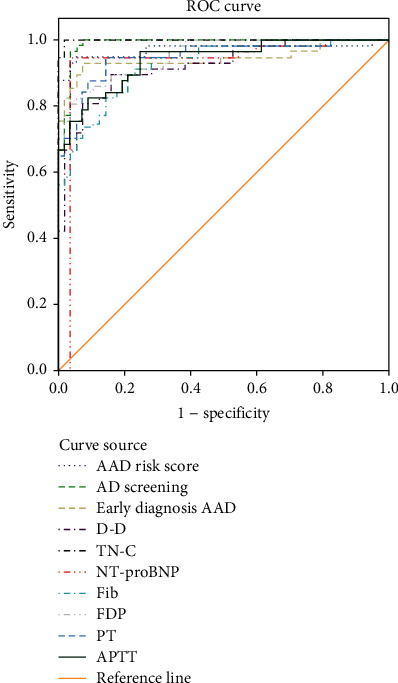
ROC curve analysis for the diagnostic value.

The three risk scores, various laboratory indicators, and various coagulation function indicators were all highly valuable in the diagnosis of acute AD (AUC > 0.9, *P* < 0.05) (Table 4 and Figure 1).

### 3.6. ROC Curve Diagnostic Efficiency

**Figure 2:**
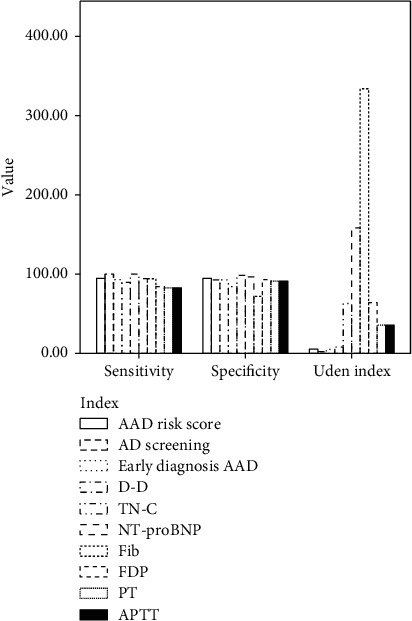
Diagnostic efficiency of each index.

Both AD screening scale and TN-C expression level had the highest sensitivity of 100% to the diagnosis of acute AD, and TN-C expression level had the highest specificity of 98.25% to the diagnosis of acute AD (Table 5 and Figure 2).

### 3.7. Multivariate Analysis

The results of the multivariate logistic regression analysis verified that Fib, FDP, PT, APTT, D-D, TN-C, and NT-proBNP were the affecting factors of AAD (*P* < 0.05, Table 6).

### 3.8. Pathology of AAD and ACS

**Figure 3:**
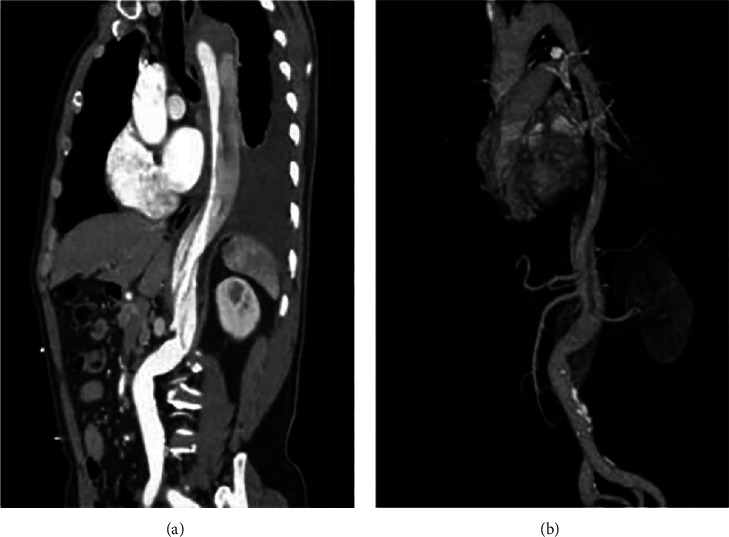
Schematic diagram showing the pathology of AAD using multi-slice spiral CT scan.

The pathological picture of AAD and ACS by multislice spiral CT scan is displayed in Figure 3. The mild coronary stenosis was 52.63% (30/57), the moderate coronary stenosis was 38.60% (22/57), and the severe coronary stenosis was 8.77% (5/57); the mild detection rate was 93.33% (28/30), the moderate detection rate was 95.45% (21/22), the severe detection rate was 100% (5/5), and the difference was statistically significant (*P* < 0.05); the positive detection rate was positively correlated with the degree of stenosis (*r* = 0.814, *P* < 0.05).

### 3.9. Thromboelastogram-Based Blood Coagulation Indicators at Various Time Points

**Figure 4:**
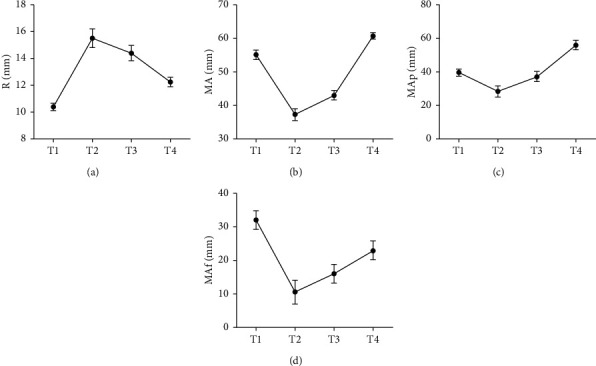
Results of thromboelastogram at different time points. Note: (a) changes in coagulation factors at different time points, (b) changes in MA at different time points, (c) changes in MAp at different time points, and (d) changes in MAf at different time points.

MA, MAf, and MAp displayed a decrease and reached the lowest point at T2. R showed an opposite trend and reached a peak point at T2. At T4, a higher MAp and a lower MAf were found than before surgery, and R and MA returned to preoperative levels (Figure 4).

## 4. Discussion

In AAD, the rupture of the aortic intima results in a false lumen that presses the true lumen in the aorta by the aggregation of blood in the aortic wall, which triggers various abnormal pathological and physiological reactions [11–13]. The manifestations of chest pain and sweating at the early stage of AAD also lead to a plight in the differentiation from acute cardiovascular diseases such as ACS [14–16]. Currently, AAD risk score, AD screening, and early diagnosis of AAD are commonly used scales to assess the risk of AD given their high sensitivity and specificity. D-D is a product of the activation of the coagulation system and the fibrinolytic system formed to dissolve the synthesis of cross-linked fibrin. It serves as an important blood coagulation system marker for the diagnosis of AAD. It has been reported that D-D has a high application value in differentiating AAD from ischemic cardiovascular and cerebrovascular diseases and vascular embolism diseases [17, 18].

TN-C, a member of the extracellular matrix tenascin family, is widely expressed in embryonic heart cells, including cells about coronary arteries, myocardium, and valves. The expression level of TN-C in the normal aortic structure is related to its borne mechanical pressure. TN-C can maintain the normal physiological functions of vascular smooth muscle cells in the aortic wall, protect the normal structure of the extracellular matrix, and inhibit a series of inflammatory reactions in the aortic wall in the onset of AAD.

AAD is associated with an aberrant increase in NT-proBNP, which may be ascribed to the left ventricular diastolic dysfunction after long-term hypertension. The onset of AAD involves coagulation and fibrinolytic system activities, with a stronger coagulation function than the fibrinolytic activity, which thus results in thrombus. Persistent fibrinolysis and coagulation can lead to the progressive consumption of coagulation factors and fibrinogen and induce diffuse intravascular coagulation.

In the present study, AD patients showed higher three risk scores, coagulation function indexes, and laboratory indexes versus the ACS patients, suggesting the feasibility of these indicators for the diagnosis of AAD. Furthermore, the ROC curve analysis revealed that all indicators had high diagnostic value, of which AD screening scale and TN-C expression level could reach a 100% sensitivity for the diagnosis of AAD, and the specificity of TN-C expression level was as high as 98.25%, suggesting a promising diagnostic value of AD, TN-C, D-D, NT-proBNP, and blood coagulation function indicators for the diagnosis of AAD. In patients with AAD, the level of coagulation privacy is lower than the normal, with a higher MA, normal MAp, and hyperactive MAf, which is attributed to the high activation of thrombin after blood contacts the false lumen of nonendothelial tissue in the early stage of the formation of aortic dissection. Therefore, the failure of the conventional blood transfusion therapy to restore a real balance of blood coagulation function in patients with AAD may be in consequence of platelet overtransfusion.

A great number of studies have confirmed that coronary angiography using DSA technology is the gold standard for diagnosing ACS and is of great significance in evaluating the degree of coronary stenosis, yet its clinical application is limited due to the invasive property [16–18]. The results of this study show that the positive detection rate of CT coronary angiography is positively correlated with the degree of stenosis, which can be used as an alternative in clinical practice. DSA and CT coronary angiography are consistent, but they have different advantages and disadvantages, so the choice of diagnostic methods should be determined according to the actual situation. Another study shows that MSCT coronary angiography is one of the most noninvasive, economical, convenient, and fast examination methods for diagnosing ACS. However, due to certain limitations, it is necessary to take the degree of coronary stenosis, risk factors, and number of diseased branches into consideration to diagnose ACS and its severity in order to improve the diagnostic efficiency [12].

In summary, AD risk scores, coagulation function index, and laboratory index are highly valuable in the diagnosis of AAD, especially the AD screening scale and TN-C expression level with high sensitivity and specificity. Additionally, other indicators are expected to improve the accuracy of diagnosis. However, this study is limited by the small number of cases included in this study, so the Youden index remains to be further verified with larger sample size.

## Figures and Tables

**Table 1:** Comparison of three risk scores between the three groups ($\bar{x}\pm s$, points).

Groups	*n*	AAD risk score	AD screening	AAD early diagnosis
ACS group	57	3.06 ± 1.33^ab^	1.30 ± 0.42^ab^	3.55 ± 1.38^ab^
AD group	57	9.68 ± 2.62^a^	2.56 ± 0.36^a^	9.31 ± 2.94^a^
Control group	50	2.15 ± 0.36	0.23 ± 0.01	2.34 ± 0.81
*F*		17.063	17.269	13.399
*P*		<0.01	<0.01	<0.01

Note: compared with control group, ^a^*P* < 0.05; compared with AD group, ^b^*P* < 0.05.

**Table 2:** Comparison of coagulation function indexes between the three groups ($\bar{x}\pm s$).

Groups	*n*	Fib (mg/dl)	FDP (*μ*g/ml)	PT (s)	APTT (s)
ACS group	57	314.95 ± 31.00^ab^	59.28 ± 10.80^ab^	13.85 ± 1.46^ab^	32.77 ± 2.46^ab^
AD group	57	382.38 ± 35.07^a^	80.91 ± 15.26^a^	14.35 ± 1.22^a^	38.84 ± 3.14^a^
Control group	50	296.14 ± 20.34	49.28 ± 9.80	11.85 ± 0.96	26.33 ± 1.16
*F*		10.877	13.171	12.210	11.474
*P*		<0.01	<0.01	<0.01	<0.01

Note: compared with control group, ^a^*P* < 0.05; compared with AD group, ^b^*P* < 0.05.

**Table 3:** Comparison of two groups of laboratory indicators ($\bar{x}\pm s\;$).

Groups	*n*	D-D (mg/l)	TN-C (*μ*g/l)	NT-proBNP (pg/ml)
ACS group	57	8.44 ± 1.63^ab^	55.71 ± 3.92^ab^	500.34 ± 40.32^ab^
AD group	57	12.37 ± 2.15^a^	69.28 ± 3.25^a^	600.48 ± 38.86^a^
Control group	50	3.36 ± 1.11	—	86.64 ± 38.38
*F*		10.984	20.139	20.162
*P*		<0.01	<0.01	<0.01

Note: compared with control group, ^a^*P* < 0.05; compared with AD group, ^b^*P* < 0.05.

**Table 4:** ROC curve analysis for the diagnostic value.

Indexes	AUC	SE	*P*	95% CI	
				Lower limit	Upper limit
AAD risk score	0.971	0.018	≤0.001	≤0.001	1.000
AD screening	0.990	0.007	≤0.001	≤0.001	1.000
AAD early diagnosis	0.945	0.024	≤0.001	0.897	0.993
D-D	0.922	0.025	≤0.001	0.874	0.971
TN-C	0.999	0.001	≤0.001	≤0.001	1.000
NT-proBNP	0.933	0.030	≤0.001	0.874	0.992
Fib	0.926	0.023	≤0.001	0.881	0.971
FDP	0.954	0.017	≤0.001	0.921	0.986
PT	0.950	0.020	≤0.001	0.910	0.989
APTT	0.940	0.021	≤0.001	0.899	0.981

**Table 5:** Diagnostic efficiency of each index.

Indexes	Sensitivity	Specificity	Youden's index
AAD risk score (points)	94.74	94.74	5.21
AD screening (points)	100.00	92.98	1.85
AAD early diagnosis (points)	92.98	92.98	5.33
D-D (mg/l)	89.47	84.21	10.14
TN-C (*μ*g/l)	100.00	98.25	62.88
NT-proBNP (pg/ml)	94.74	96.49	158.05
Fib (mg/dl)	94.74	71.93	333.72
FDP (*μ*g/ml)	84.21	92.98	63.64
PT (s)	82.46	91.23	35.70
APTT (s)	82.46	91.23	35.70

**Table 6:** Multivariate result analysis.

Exposure factors	Univariate analysis OR (95% CI)	*P* value	Multivariate result analysis OR (95% CI)	
			Correction model	*P* value
Fib	0.926 (0.076, 1.116)	<0.01	0.812 (0.080, 1.056)	0.035
FDP	0.954 (0.211, 1.541)	<0.01	0.914 (0.720, 1.116)	0.011
PT	0.95 (0.161, 1.046)	<0.01	0.934 (0.120, 1.235)	0.001
APTT	0.94 (0.521, 1.746)	<0.01	0.911 (0.230, 1.636)	0.002
D-D	0.922 (0.331, 1.216)	<0.01	0.896 (0.442, 1.650)	0.035
TN-C	0.999 (0.631, 1.336)	<0.01	0.91 (0.552, 1.226)	0.022
NT-proBNP	0.933 (0.551, 1.236)	<0.01	0.874 (0.123, 1.142)	0.001

## Data Availability

The datasets used and analyzed during the current study are available from the corresponding author on reasonable request.
